# Free Thyroxine Level as an Independent Predictor of Infection-Related Mortality in Patients on Peritoneal Dialysis: A Prospective Multicenter Cohort Study

**DOI:** 10.1371/journal.pone.0112760

**Published:** 2014-12-01

**Authors:** Hee-Yeon Jung, Jang-Hee Cho, Hye Min Jang, Yon Su Kim, Shin-Wook Kang, Chul Woo Yang, Nam-Ho Kim, Ji-Young Choi, Sun-Hee Park, Chan-Duck Kim, Yong-Lim Kim, Clinical Research Center for End Stage Renal Disease (CRC for ESRD) Investigators

**Affiliations:** 1 Department of Internal Medicine, Kyungpook National University School of Medicine, Daegu, Korea; 2 Department of Statistics, Kyungpook National University, Daegu, Korea; 3 Department of Internal Medicine, Seoul National University College of Medicine, Seoul, Korea; 4 Department of Internal Medicine, Yonsei University College of Medicine, Seoul, Korea; 5 Department of Internal Medicine, The Catholic University of Korea College of Medicine, Seoul, Korea; 6 Department of Internal Medicine, Chonnam National University Medical School, Gwangju, Korea; 7 Clinical Research Center for End Stage Renal Disease, Daegu, Korea; 8 Bk21 Plus KNU Biomedical Convergence Program, Department of Biomedical Science, Kyungpook National University, Daegu, Korea; Sao Paulo State University, Brazil

## Abstract

**Background:**

Previous studies have reported the relationship between thyroid hormone levels and mortality in dialysis patients. However, little is known about the association of free thyroxine (fT4) and mortality in patients on peritoneal dialysis (PD). This study investigated the association between basal and annual variation in fT4 level and mortality in PD patients.

**Methods:**

Patients on maintenance PD were enrolled from a prospective multicenter cohort study in Korea; their serum triiodothyronine, fT4, and thyroid-stimulating hormone levels were measured 12 months apart. Patients with overt thyroid disease and those receiving thyroid hormone replacement therapy were excluded from the analysis. Patients were divided into two groups based on the median levels of fT4. The differences of all-cause, infection-related, and cardiovascular mortalities were analyzed between the two groups. The association of basal levels and annual variation with mortality was investigated with Kaplan–Meier curves and Cox proportional hazard models.

**Results:**

Among 235 PD patients, 31 (13.2%) deaths occurred during the mean follow-up period of 24 months. Infection (38.7%) was the most common cause of death. Lower basal fT4 levels were an independent predictor of all-cause and infection-related death (hazard ratio [HR] = 2.74, 95% confidence interval [CI] 1.27–5.90, *P* = 0.01, and HR = 6.33, 95% CI 1.16–34.64, *P* = 0.03, respectively). Longitudinally, patients with persistently lower fT4 levels during the 12-month period had significantly higher all-cause mortality than those with persistently higher levels (HR = 3.30, 95% CI 1.15–9.41, *P* = 0.03). The area under the receiver operating characteristic curve of fT4 for predicting all-cause and infection-related mortality was 0.60 and 0.68, respectively.

**Conclusions:**

fT4 level is an independent predictor of mortality and is especially attributable to infection in PD patients. This predictor was consistent when considering both baseline measurements and annual variation patterns. Close attention to infection in PD patients with relatively lower fT4 levels should be considered.

## Introduction

End-stage renal disease (ESRD) is a frequent cause of altered thyroid hormone levels in the absence of underlying intrinsic thyroid disorder. A variety of alterations in thyroid hormone levels and/or metabolism has been described in patients with ESRD, ranging from low total and free triiodothyronine (T3) and thyroxine (T4) levels to subclinical and clinical hypothyroidism [1]. Several studies suggested that as many as 70% of patients with ESRD presented with low T3 levels and as many as 20–25% had subclinical hypothyroidism [2], [3]. These alterations reflects iodine retention, diminished conversion of T4 to T3 in the periphery, reduced serum protein-binding capacity [1], and metabolic acidosis [4] in patients with ESRD. These derangements have long been considered an innocent metabolic adaptation to a chronic condition that causes energy and protein wasting [5]. However, recent reports about dialysis patients have suggested that low T3 levels and subclinical hypothyroidism are associated with higher cardiovascular and all-cause mortality [5]–[8].

Although free thyroxine (fT4) level is regarded an important determination of thyroid status, little is known about its association with mortality in dialysis patients. Some studies have revealed that low T4 levels were associated with higher mortality rates in nonrenal patients with nonthyroidal illness [9], [10] and in hemodialysis (HD) patients [6]. However, the impact of fT4 as a mortality factor in euthyroid patients on peritoneal dialysis (PD) has not been clearly defined. Moreover, it is not known whether fT4 level fluctuation over time affects mortality hazards in PD patients. This study investigated the association between basal and annual variation of fT4 levels and mortality in PD patients.

## Materials and Methods

### Patients

A total of 235 maintenance PD patients were enrolled from a prospective multicenter cohort study in Korea. Patients with overt hypothyroidism or overt hyperthyroidism were excluded. Overt hypothyroidism was defined as thyroid-stimulating hormone (TSH) level>10 mIU/L with decreased fT4 level; overt hyperthyroidism was defined as the TSH level <0.1 mIU/L with increased fT4 level. Patients who received thyroid hormone replacement therapy and other drugs that might interfere with thyroid function, such as lithium and amiodarone, were also excluded. All patients provided written informed consent before inclusion and the Institutional Review Board of each center approved the study protocol. [The Catholic University of Korea, Bucheon St. Mary's Hospital; The Catholic University of Korea, Incheon St. Mary's Hospital; The Catholic University of Korea, Seoul St. Mary's Hospital; The Catholic University of Korea, St. Mary's Hospital; The Catholic University of Korea, St. Vincent's Hospital; The Catholic University of Korea, Uijeongbu St. Mary's Hospital; Cheju Halla General Hospital; Chonbuk National University Hospital; Chonnam National University Hospital; Chung-Ang University Medical Center; Chungbuk National University Hospital; Chungnam National University Hospital; Dong-A University Medical Center; Ehwa Womens University Medical Center; Fatima Hospital, Daegu; Gachon University Gil Medical Center; Inje University Pusan Paik Hospital; Kyungpook National University Hospital; Kwandong University College of Medicine, Myongji Hospital; National Health Insurance Corporation Ilsan Hospital; National Medical Center; Pusan National University Hospital; Samsung Medical Center, Seoul; Seoul Metropolitan Government, Seoul National University, Boramae Medical Center; Seoul National University Hospital; Seoul National University, Bundang Hospital; Yeungnam University Medical Center; Yonsei University, Severance Hospital; Yonsei University, Gangnam Severance Hospital; Ulsan University Hospital; Wonju Christian Hospital (in alphabetical order)]. All clinical investigations were conducted in accordance with the guidelines of the 2008 Declaration of Helsinki.

### Follow-up and Outcome Ascertainment

The follow-up period was May 2009–December 2011, and patients were followed up for the occurrence of death. Mortality was classified as all-cause, infection-related, and cardiovascular mortality. Cardiovascular mortality was defined as death from myocardial infarction, arrhythmia, or stroke, or sudden death. During the mean follow-up period of 24 months, serum T3, fT4, and TSH levels were measured 12 months apart. fT4 (reference range, 0.8 to 1.8 nmol/L) and T3 levels (reference range, 0.6 to 1.9 nmol/L) were measured by commercially available radiommunoassay kits (Beckman Coulter, Czech Republic and Cisbio Bioassays, France, respectively) and TSH level (reference range, 0.3 to 4.0 mIU/L) by an immunoradiometric assay (Brahms, Germany). Patients were divided into two groups dichotomized based on the median levels of baseline fT4 and on annual variations of fT4 level. To analyze annual fT4 level variations, we classified patients according to their variation along the median distribution at baseline and 12-month follow-up and labeled individuals in either a “persistently high” group or a “persistently low” group. All-cause, infection-related, and cardiovascular mortalities were compared between the two groups.

### Statistical analysis

All data were expressed as medians with ranges or mean ± SD. Differences between groups were tested by independent sample *t*-tests and chi-squared tests as appropriate. Survival rate during follow-up was analyzed by the Kaplan–Meier method, and hazard ratios (HRs) were calculated with Cox proportional hazard models with different degrees of adjustment. Age, sex, comorbidities, serum hemoglobin, serum albumin, low-density lipoprotein, serum C-reactive protein, blood pressure, peritonitis rate, estimated glomerular filtration rate (eGFR) at baseline, weekly KT/V, PD modality, and use of vitamin D were considered possible confounders. The ability of fT4 to predict mortality was analyzed further with receiver operating characteristic (ROC) curves. The statistical analysis was performed using SAS system for Windows, version 9.2 (SAS Institute Inc., Cary, NC) and R (R Foundation for Statistical Computing, Vienna, Austria; www.r-project.org). *P* values <0.05 were considered statistically significant.

## Results

### Patient characteristics and thyroid function test

The patients' main demographic, clinical, and biochemical characteristics are detailed in Table 1. The mean age of the subjects was 51.4 years, and 56.2% were men. Diabetes was the most common cause of ESRD (38.2%), followed by glomerulonephritis (33.8%) and hypertension (21.1%). Twenty-four patients (11.8%) had the comorbidity of cerebrovascular disease, 23 patients (11.3%) had congestive heart failure, and 21 patients (10.3%) had coronary artery disease.

**Table 1:** Baseline demographic, clinical, and biochemical data according to fT4 dichotomization in 235 peritoneal dialysis patients.

	Total (n = 235)	fT4≥1.1 ng/mL (n = 152)	fT4<1.1 ng/mL (n = 83)	*P* value
Age (years)	51.4±13.3	51.6±12.9	50.9±13.9	0.68
Sex (% men)	132 (56.2)	93 (61.2)	39 (47.0)	0.04
Body mass index (kg/m^2^)	22.6±3.1	22.7±3.2	22.3±2.9	0.27
Dialysis duration (months)	65.1±51.8	66.1±53.4	63.4±49.1	0.71
Primary renal disease, n (%)				
Diabetes	87 (38.2)	64 (43.8)	23 (28.1)	0.11
Hypertension	48 (21.1)	26 (17.8)	22 (26.8)	
Glomerulonephritis	77 (33.8)	46 (31.5)	31 (37.8)	
Others	16 (7.0)	10 (6.9)	6 (7.3)	
Comorbidity, n (%)				
Congestive heart failure	23 (11.3)	14 (10.5)	9 (13.0)	0.58
Coronary artery disease	21 (10.3)	16 (11.9)	5 (7.3)	0.30
Peripheral vascular disease	5 (2.5)	3 (2.3)	2 (2.9)	1.00
Arrhythmia	2 (1.0)	1 (0.8)	1 (1.5)	1.00
Cerebrovascular disease	24 (11.8)	19 (14.2)	5 (7.3)	0.15
Chronic lung disease	9 (4.5)	7 (5.3)	2 (2.9)	0.72
Peptic ulcer disease	10 (4.9)	5 (3.7)	5 (7.3)	0.31
Moderate-severe liver disease	8 (4.0)	3 (2.2)	5 (7.4)	0.12
Connective tissue disease	18 (8.9)	12 (9.0)	6 (8.7)	0.95
Tumor	3 (1.5)	2 (1.5)	1 (1.55)	1.00
Smokers, n (%)				
Nonsmoker	148 (63.5)	85 (56.7)	63 (75.9)	0.01
Smoker	18 (7.7)	14 (9.3)	4 (4.8)	
Ex-smoker	67 (28.8)	51 (34.0)	16 (19.3)	
Laboratory data				
Hemoglobin (g/dL)	8.9±1.8	9.9±1.8	9.9±1.9	0.93
Albumin (g/dL)	3.6±0.5	3.7±0.5	3.6±0.5	0.33
Calcium (mg/dL)	8.2±1.0	8.2±1.1	8.2±1.0	0.76
Phosphate (mg/dL)	4.9±1.5	5.0±1.6	4.8±1.3	0.27
LDL (mg/dL)	103.4±30.8	100.4±30.8	108.9±30.3	0.04
Triglycerides (mg/dL)	141.7±96.8	146.0±105.2	133.7±78.9	0.31
Total cholesterol (mg/dL)	171.4±39.0	168.6±40.7	176.6±35.4	0.13
Ferritin (ng/mL)	178.8±167.6	168.3±159.7	198.1±180.6	0.19
CRP (mg/dL)	0.8±2.0	0.9±2.2	0.7±1.5	0.34
Baseline T3 (nmol/L)	0.87±0.30	0.87±0.28	0.87±0.32	0.95
Baseline TSH (mIU/L)	3.10±2.24	2.94±2.07	3.40±2.52	0.15
Baseline eGFR (mL/min/1.73 m^2^)	6.1±4.4	6.4±5.1	5.4±2.3	0.08
Peritonitis rate (episode/patient-year)	0.22	0.22	0.22	0.83
Weekly KT/V	2.1±1.4	2.2±1.6	2.0±0.7	0.21
Peritoneal dialysis modality				
CAPD	202 (86.0)	133 (87.5)	69 (83.1)	0.34
APD	33 (14.0)	19 (12.5)	14 (16.9)	
Use of vitamin D	47 (20.0)	29 (19.1)	18 (21.7)	0.63

Values are shown as mean ± standard deviation.

APD, automated peritoneal dialysis; CAPD, continuous ambulatory peritoneal dialysis; CRP, C-reactive protein; eGFR, estimated glomerular filtration rate; LDL, low-density lipoprotein; T3, triiodothyronine; TSH, thyroid-stimulating hormone.

In all patients, the baseline levels of T3 (reference range: 0.6–1.9 nmol/L) and TSH (reference range: 0.3–4.0 mIU/L) were 0.87±0.30 nmol/L and 3.10±2.24 mIU/L, respectively. Baseline level of fT4 (reference range: 0.8–1.8 nmol/L) was 1.16±0.26 nmol/L. Among the enrolled patients, 192 (81.7%) were euthyroid, 38 (16.2%) had subclinical hypothyroidism, and 5 (2.1%) had subclinical hyperthyroidism.

When patients were divided into the two groups on the basis of median fT4 concentrations (1.1 ng/mL), there were no significant differences in comorbidity, dialysis duration, body mass index, peritonitis rate, PD modality, or use of vitamin D between the two groups. In addition, serum hemoglobin, serum albumin, serum C-reactive protein, ferritin, lipid profiles, eGFR at baseline, and weekly KT/V were not different between the groups. However, patients with relatively lower fT4 levels were more often women, nonsmokers, and those with increased low-density lipoprotein levels (Table 1).

### Basal fT4 level and its association with mortality

Among the 235 PD patients, 31 (13.2%) deaths occurred during the mean follow-up period of 24 months. Infection (38.7%) was the most common cause of death, followed by cardiovascular disease (19.4%) (Table 2).

**Table 2:** Cause of death in the study cohort.

Causes of death	n (%)
Infection-related	12 (38.7)
Peritonitis	5 (16.1)
Pneumonia	3 (9.7)
Sepsis	2 (6.5)
Urinary tract infection	2 (6.5)
Cardiovascular	6 (19.4)
Sudden death	3 (9.7)
Myocardial infarction	1 (3.2)
Arrhythmia	1 (3.2)
Stroke	1 (3.2)
Gastrointestinal hemorrhage	3 (9.7)
Cachexia	1 (3.2)
Others	2 (6.5)
Unknown	7 (22.6)
Total	31 (100.0)

**Figure 1:**
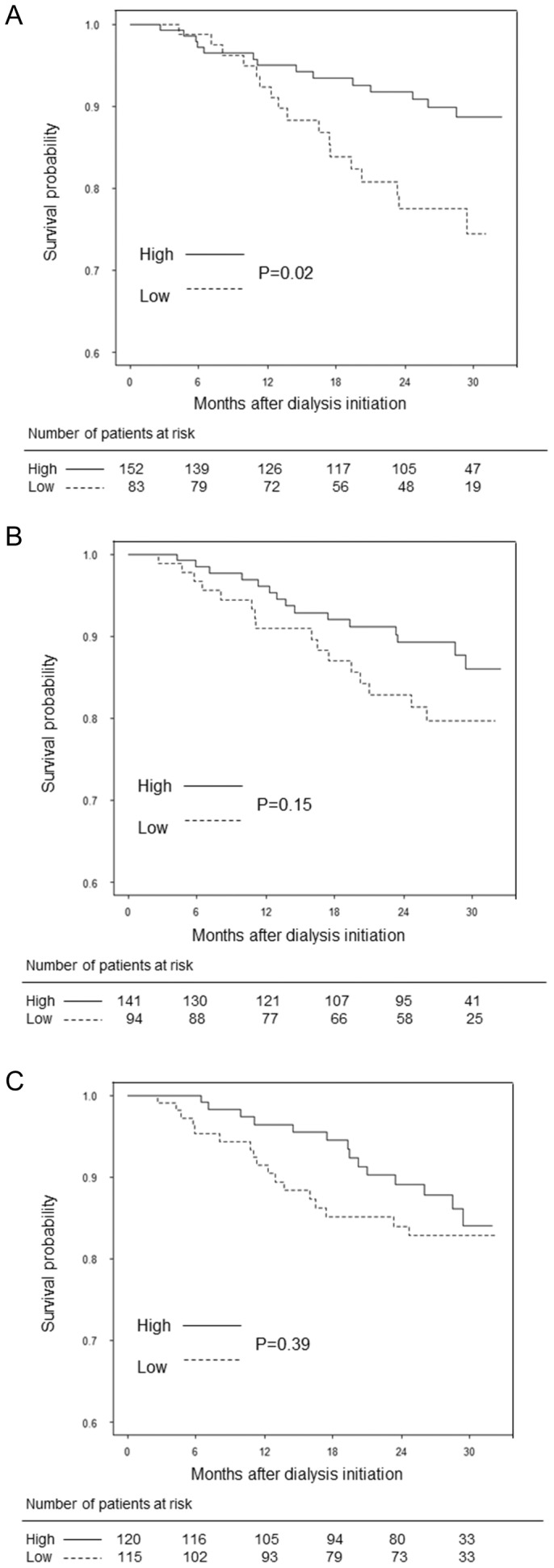
Kaplan–Meier survival curves for fT4 (A), T3 (B), and TSH (C). In a Kaplan–Meier analysis, lower fT4 levels were associated with death (*P* = 0.02), but neither T3 nor TSH levels were associated with death (*P* = 0.15 and *P* = 0.39, respectively). fT4, free thyroxine; T3, triiodothyronine; TSH, thyroid-stimulating hormone.

In a Kaplan–Meier analysis, lower fT4 levels were associated with death (*P* = 0.02), but neither T3 nor TSH were associated with death (*P* = 0.15 and *P* = 0.39, respectively) (Figure 1). As shown in Table 3, the crude HR of all-cause mortality was 2.35 (95% confidence interval [CI], 1.16–4.78) in patients with lower fT4 levels (*P* = 0.02). After adjusting for confounders (age, sex, diabetes, systolic pressure, diastolic pressure, hemoglobin, albumin, C-reactive protein, low-density lipoprotein, peritonitis rate, eGFR at baseline, weekly KT/V, PD modality, and use of vitamin D), the adjusted HR of all-cause mortality was 2.74 (95% CI, 1.27–5.90) in patients with lower fT4 levels (*P* = 0.01).

**Table 3:** Mortality HRs according to baseline fT4 level in 235 peritoneal dialysis patients.

Parameter	Crude		Model 1^a^		Model 2^b^	
	HR (95% CI)	*P* value	HR (95% CI)	*P* value	HR (95% CI)	*P* value
fT4<1.1 ng/mL						
All-cause mortality	2.35 (1.16–4.78)	0.02	2.34 (1.15–4.77)	0.02	2.74 (1.27–5.90)	0.01
Infection-related mortality	3.89 (1.17–12.95)	0.03	3.61 (1.08–12.13)	0.04	6.33 (1.16–34.64)	0.03
Cardiovascular mortality	3.69 (0.68–20.17)	0.13	4.85 (0.85–27.73)	0.08	7.78 (1.00–60.40)	0.05

95% CI, 95% confidence interval; HR, hazard ratio.

a Model 1: Adjusted for age and sex.

b Model 2: Adjusted for age, sex, diabetes, systolic pressure, diastolic pressure, hemoglobin, albumin, C-reactive protein, low-density lipoprotein, peritonitis rate, estimated glomerular filtration rate at baseline, weekly KT/V, peritoneal dialysis modality, and use of vitamin D.

**Figure 2:**
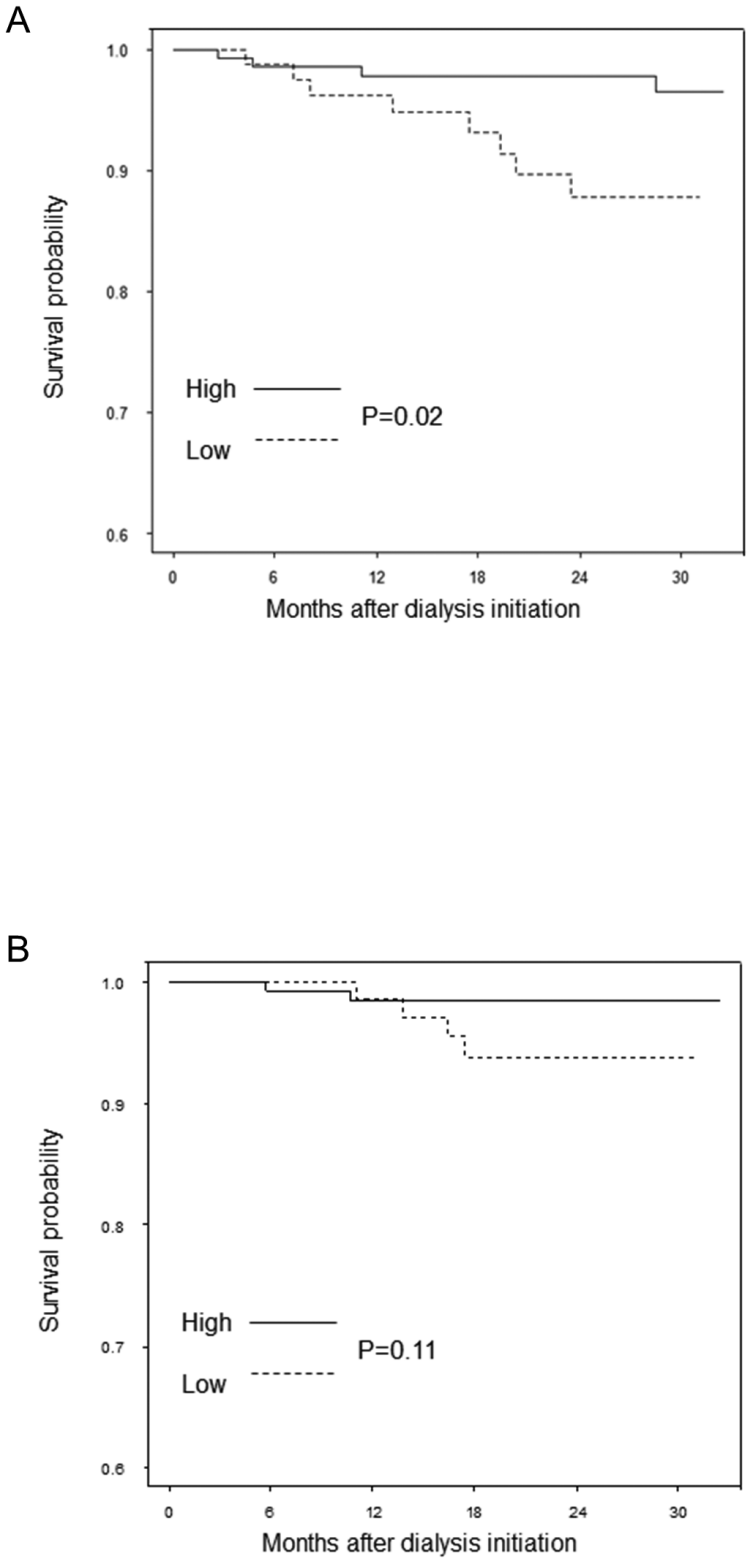
Kaplan–Meier curves for infection-related (A) and cardiovascular (B) deaths. In a Kaplan–Meier analysis, lower fT4 levels were also associated with infection-related deaths (*P* = 0.02). Contrary to the infection-related deaths, lower fT4 levels were not significantly associated with cardiovascular deaths in a Kaplan–Meier analysis (*P* = 0.11). fT4, free thyroxine.

The differences in infection-related and cardiovascular mortalities between the two groups were also analyzed. In a Kaplan–Meier analysis, lower fT4 levels were associated with infection-related deaths (*P* = 0.02) (Figure 2). HRs of infection-related deaths were considerably higher in magnitude (crude HR, 3.89 [95% CI, 1.17–12.95], *P* = 0.03) and remained so after adjustment for confounders (adjusted HR, 6.33 [95% CI, 1.16–34.64], *P* = 0.03) (Table 3).

A similar analysis was performed for cardiovascular deaths. Contrary to the infection-related deaths, lower fT4 levels were not significantly associated with cardiovascular deaths in a Kaplan–Meyer analysis (*P* = 0.11) (Figure 2). The Cox proportional hazard model also showed that lower fT4 levels were not significantly associated with cardiovascular mortalities (crude HR, 3.69 [95% CI, 0.68–20.17], *P* = 0.13; adjusted HR, 7.78 [95% CI 1.00–60.40], *P* = 0.05, respectively) (Table 3).

**Figure 3:**
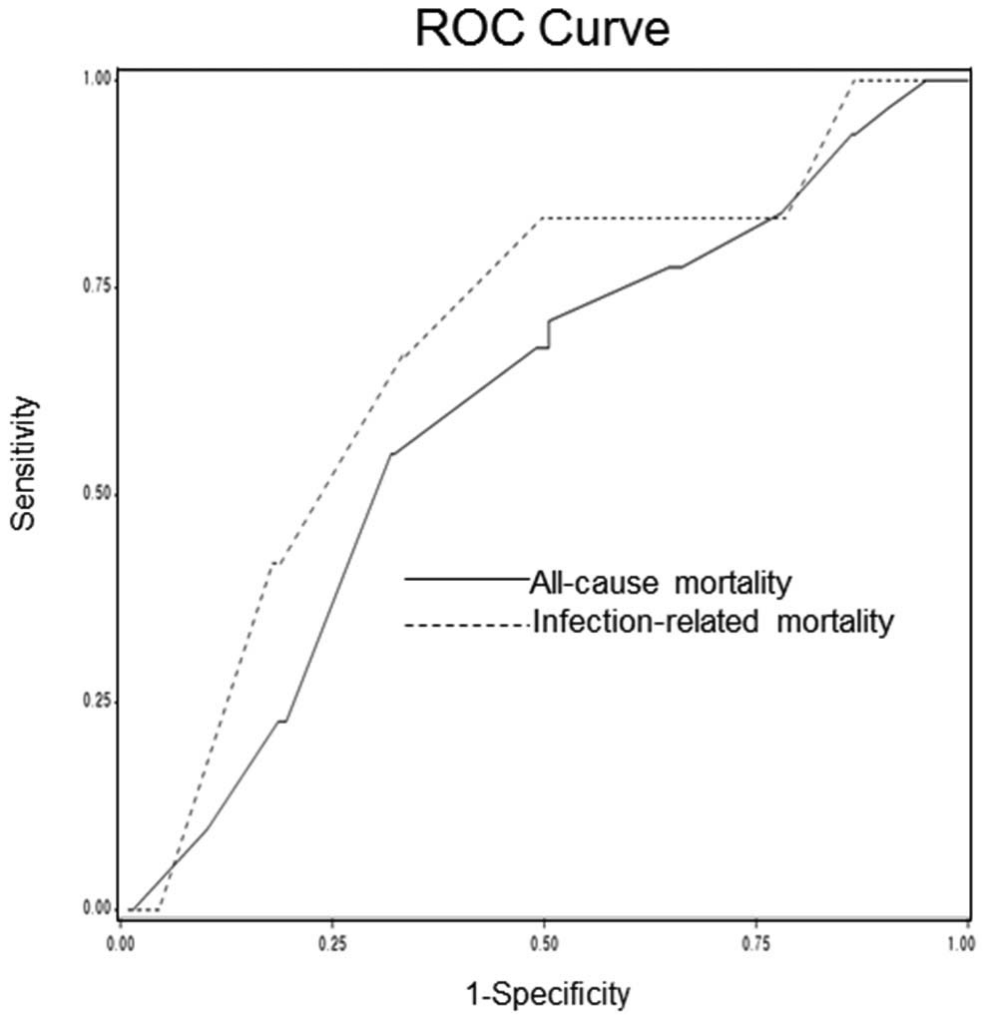
ROC curve for fT4 levels. The areas under the curve of fT4 for predicting all-cause and infection-related mortalities were 0.602 and 0.681, respectively. A cutoff fT4 level of 1.1 ng/mL had a sensitivity and specificity of 67.7% and 51.0%, respectively, for predicting all-cause mortality. A cutoff fT4 level of 1.1 ng/mL had a higher sensitivity and similar specificity of 83.3% and 50.2%, respectively, for predicting infection-related mortality than for all-cause mortality. fT4, free thyroxine; ROC, receiver operating characteristic.

ROC curve analysis for predicting all-cause mortality showed that sensitivity and specificity of a cutoff fT4 of 1.1 ng/mL were 67.7% and 51.0%, respectively. A cutoff fT4 level of 1.1 ng/mL had a higher sensitivity and similar specificity of 83.3% and 50.2%, respectively, for predicting infection-related mortality than for all-cause mortality. The areas under the curves of fT4 for predicting all-cause and infection-related mortalities were 0.602 and 0.681, respectively, demonstrating that fT4 level could be considered as a fair predictor of infection-related mortality [11] (Figure 3).

### fT4 level annual variation and its association with mortality

Thyroid hormone levels were assessed again after 12 months in all patients. Individuals with both values (baseline and 12-month follow-up) within the higher median of distribution were considered the persistently high group; those with both values within the lower median of distribution were considered the persistently low group. General characteristics are depicted across two different fT4 level variation groups, and no major differences are observed between these groups (Table S1). There were also no significant differences in the baseline and 12-month follow-up levels of T3 and TSH between the two groups (Table S1). Patients with persistently lower fT4 levels had significantly higher all-cause mortality than those with persistently higher levels after adjusting for confounders (adjusted HR, 3.30 [95% CI 1.15–9.41], *P* = 0.03). Persistently lower fT4 levels showed, albeit not significantly, a trend for increased infection-related mortality (Table 4).

**Table 4:** Mortality HRs according to fT4 level annual variation.

Parameter	Crude		Model 1^a^		Model 2^b^	
	HR (95% CI)	*P* value	HR (95% CI)	*P* value	HR (95% CI)	*P* value
Persistently low						
All-cause mortality	2.20 (0.89–5.47)	0.09	2.39 (0.93–6.15)	0.07	3.30 (1.15–9.41)	0.03
Infection-related mortality	6.24 (0.70–55.84)	0.10	6.98 (0.76–63.74)	0.11	12.42 (0.68–226.67)	0.09
Cardiovascular mortality	3.20 (0.29–35.32)	0.34	5.47 (0.42–71.44)	0.20	5.42 (0.22–128.75)	0.30

95% CI, 95% confidence interval; HR, hazard ratio.

a Model 1: Adjusted for age and sex.

b Model 2: Adjusted for age, sex, diabetes, systolic pressure, diastolic pressure, hemoglobin level, albumin level, C-reactive protein level, low-density lipoprotein, peritonitis rate, estimated glomerular filtration rate at baseline, weekly KT/V, peritoneal dialysis modality, and use of vitamin D.

## Discussion

This multicenter prospective cohort study shows the association of low fT4 levels with higher mortality rates in maintenance PD patients. Longitudinal low fT4 levels as well as baseline low fT4 levels are significant independent risk factors for mortality in PD patients. In addition, we demonstrated that baseline low fT4 level was an independent predictor of infection-related mortality. To the best of our knowledge, this is the first study to report that baseline and annual variation of fT4 levels are related with mortality in PD patients.

The most common alteration in thyroid hormone level in a dialysis population is low T3 level regardless of serum TSH levels. Advanced stage of chronic kidney disease (CKD) showed a high prevalence of low T3 level, with as many as 78.6% among the participants having estimated glomerular filtration rates (GFR) <15 mL/min/1.73 m^2^ [2]. The patients with low T3 levels were associated with an inflammatory state [8], [12], endothelial dysfunction [13]–[15], atherosclerotic tendency [13], and ventricular dysfunction [16], [17]. Several studies have reported the association of low T3 levels and subclinical hypothyroidism with mortality in CKD patients, suggesting that a low T3 level was a consistent predictor of all-cause and cardiovascular death in predialysis CKD [18], HD [5], [6], and PD [8] patients. Subclinical hypothyroidism is another common abnormality in CKD patients. The prevalence of hypothyroidism was reported to increase as the estimated GFR decreased [3]. Subclinical hypothyroidism could induce depression of left ventricular systolic function [19], alteration in flow-mediated vasodilation [20], and increased arterial stiffness [21]. Based on the association between hypothyroidism and cardiovascular disease, recent data have indicated that hypothyroidism independently predicted higher mortality rates in ESRD patients [7], [22].

Although the relationships of T3 and TSH levels with mortality have been reported in dialysis patients, the association of fT4 level and mortality in dialysis patients is controversial. Previous studies reported that there was no significant association between fT4 level and death in predialysis [18] and HD [5] patients. However, a recent study with 210 HD patients revealed that low T4 levels were associated with higher mortality rates caused by cardiovascular disease [6]. Longitudinal low T4 levels also showed an increased risk of all-cause mortality and that the association was not altered after adjustment for T3 level and other confounders. Little is known about the impact of fT4 level on mortality in PD patients. One study of 41 PD patients showed no significant difference in fT4 level between survivors and nonsurvivors [8], but this small study was underpowered to assess the independent association of fT4 level and mortality using multivariate analysis. Another single-center study that enrolled 278 PD patients also did not find a significant relationship between time-dependent fT4 levels and cardiovascular mortality. The retrospective study design and enrollment period of>30 years may have reduced the statistical power in that study [23].

The current study analyzed the impact of fT4 level on mortality in maintenance PD patients and showed that low fT4 level independently predicted increased risk of mortality. We enrolled a moderate number of patients from a multicenter cohort and prospectively followed patient survival. Contrary to the previous studies with ESRD patients, T3 and TSH levels were not associated with mortality in our study. Considering that the other studies mainly targeted HD patients [5]–[7], [22], [24], the difference might be related to modality effect of the thyroid function test. Frequent heparinization in HD patients interfered with the fT3 assay [25] and transiently elevated fT4 levels [26]. In PD patients, peritoneal losses of thyroid-binding globulin could affect T3 and TSH levels [27]. In addition, the iodine used as a disinfectant in the PD cap has been reported to suppress thyroid hormone synthesis [28]. Because dialysis could affect thyroid function test results, clinical predictability of each thyroid hormone might be different according to dialysis modality. Given that fT4 of PD patients—rather than T3 and TSH levels—was associated with mortality in our study, fT4 level could be a more sensitive marker to predict mortality in PD patients than other thyroid hormones.

Previous studies mostly focused on the effect of abnormal thyroid function on cardiovascular outcomes [6], [18], [22], [23]. Cardiovascular disease was reported to be a leading cause of death in the ESRD patients from other cohorts [29], [30]. In contrast, a Korean ESRD cohort was distinguished from other cohorts in that the prevalence of cardiovascular disease was relatively lower and infections were the most common cause of death [31]. In the general population, subclinical thyroid patients with high cardiovascular risk were associated with a higher mortality risk [32]–[34], whereas patients with an average cardiovascular risk did not show such a relationship [35], [36]. Given the discrepant impact of thyroid disease on mortality, the slight association of abnormal thyroid function and cardiovascular mortality might be explainable in our patients who had a relatively lower risk of cardiovascular disease.

Another notable aspect of this study is that lower fT4 level was a highly significant predictor of infection-related mortality in PD patients. Baseline low fT4 levels were associated with all-cause and infection-related mortality but not with cardiovascular mortality. Patients with persistently low fT4 levels showed significantly increased all-cause mortality and trend for increased infection-related mortality. This means that the association of fT4 level with all-cause mortality was attributable to infection-related mortality. The underlying pathogenesis for the association of low T4 level with all-cause and infection-related mortality is unknown. Alteration in thyroid hormone levels occurs in patients with systemic nonthyroidal disease who appear clinically euthyroid [37]. Serum T4 concentration may also be decreased in patients with severe illnesses [38]. In experimental modes, there was a transient drop in circulating T4 levels after systemic exposure to natural or synthetic antigens [39]–[41]. In addition, T4 and T3 levels have been reported to have a differential effect on immune cells: T4 stimulated peripheral lymphocyte proliferation, whereas T3 inhibited the immune cell proliferation [42]. We were unable to differentiate whether low T4 level was a representation of the underlying condition or a risk factor modulating the immune system. Nevertheless, our findings suggest that the resulting low T4 level, whatever the causality, persisted in a considerable number of patients because the majority (75.9%) of patients with low fT4 level remained in the low fT4 level group at the annual follow-up, which in turn could affect patient mortality. To determine the pathophysiologic mechanisms of low T4 level, further studies are warranted that would evaluate several cytokines, including tumor necrosis factor-α and interleukin-lβ, which play several important roles in modulating the immune–thyroid system in vitro and in vivo [43]–[45].

Our study has some limitations. First, the duration of follow-up in this cohort study is relatively short. Additional extensive studies with longer duration of follow-up are required to confirm the association between fT4 level and mortality. Second, we could not demonstrate the effect of thyroid hormone replacement in PD patients with lower fT4 level. Intervention studies are needed to determine whether thyroid hormone supplementation improves the survival of PD patients with lower fT4 level. Third, we could not explain the causality between lower fT4 level and mortality. To determine the causality, further studies evaluating immune–thyroid system are needed.

In conclusion, fT4 levels are an independent predictor of mortality that are especially attributable to infection in PD patients. This was consistent when considering both baseline measurements and annual variation patterns. Close attention to infection in PD patients with relatively lower fT4 levels should be considered.

## Supporting Information

Table S1 **General characteristics according to fT4 level annual variation.**
(DOCX)
