# Supplementary material for: Free Thyroxine Level as an Independent Predictor of Infection-Related Mortality in Patients on Peritoneal Dialysis: A Prospective Multicenter Cohort Study
Source: PLoS One. 2014 Dec 1;9(12):e112760. doi: 10.1371/journal.pone.0112760 (PMC4249823; doi:10.1371/journal.pone.0112760)
Supplement: Table S1 — General characteristics according to fT4 level annual variation. (DOCX) [file pone.0112760.s001.docx]

**Table S1. General characteristics according to fT4 level annual variation.**

|  | Persistently high  (n = 103) | Persistently low  (n = 63) | *P* value |
| --- | --- | --- | --- |
| Age (years) | 52.6 ± 13.3 | 50.9 ± 13.8 | 0.42 |
| Sex (% men) | 60 (58.3) | 30 (47.6) | 0.18 |
| Body mass index (kg/m^2^) | 22.7 ± 3.3 | 22.6 ± 2.9 | 0.75 |
| Dialysis duration (months) | 68.4 ± 58.3 | 65.6 ± 49.3 | 0.75 |
| Primary renal disease, n (%) |  |  |  |
| Diabetes | 43 (43.9) | 16 (25.8) | 0.06 |
| Hypertension | 13 (13.3) | 17 (27.4) |  |
| Glomerulonephritis | 35 (35.7) | 24 (38.7) |  |
| Others | 7 (7.1) | 5 (8.1) |  |
| Comorbidity, n (%) |  |  |  |
| Congestive heart failure | 12 (12.5) | 7 (13.7) | 0.83 |
| Coronary artery disease | 13 (13.5) | 3 (5.9) | 0.16 |
| Peripheral vascular disease | 3 (3.2) | 2 (3.9) | 1.00 |
| Arrhythmia | 0 (0.0) | 1 (2.0) | 0.35 |
| Cerebrovascular disease | 11 (11.5) | 4 (7.8) | 0.49 |
| Chronic lung disease | 6 (6.3) | 2 (3.9) | 0.71 |
| Peptic ulcer disease | 4 (4.2) | 3 (5.9) | 0.69 |
| Moderate-severe liver disease | 3 (50.0) | 3 (50.0) | 0.15 |
| Connective tissue disease | 9 (9.4) | 5 (9.8) | 1.00 |
| Tumor | 2 (2.1) | 1 (2.0) | 1.00 |
| Smokers, n (%) |  |  |  |
| Nonsmoker | 54 (53.5) | 49 (77.8) | 0.007 |
| Smoker | 10 (9.9) | 3 (4.8) |  |
| Ex-smoker | 37 (36.6) | 11 (17.5) |  |
| Laboratory data |  |  |  |
| Hemoglobin (g/dL) | 9.8 ± 1.8 | 10.1 ± 1.9 | 0.40 |
| Albumin (g/dL) | 3.6 ± 0.5 | 3.6 ± 0.5 | 0.97 |
| Calcium (mg/dL) | 8.1 ± 1.0 | 8.2 ± 1.1 | 0.49 |
| Phosphate (mg/dL) | 5.0 ± 1.6 | 4.9 ± 1.3 | 0.67 |
| LDL (mg/dL) | 99.1 ± 33.2 | 107.9 ± 30.5 | 0.09 |
| Triglycerides (mg/dL) | 145.7 ± 95.5 | 127.7 ± 74.9 | 0.18 |
| Total cholesterol (mg/dL) | 166.5 ± 41.8 | 176.1 ± 34.4 | 0.11 |
| Ferritin (ng/mL) | 155.7 ± 149.0 | 206.1 ± 184.4 | 0.07 |
| CRP (mg/dL) | 1.0 ± 2.5 | 0.6 ± 0.4 | 0.17 |
| Baseline T3 (nmol/L) | 0.85 ± 0.29 | 0.88 ± 0.33 | 0.45 |
| Baseline fT4 (nmol/L) | 1.32 ± 0.20 | 0.91 ± 0.11 | <0.001 |
| Baseline TSH (mIU/L) | 2.91 ± 2.10 | 3.50 ± 2.60 | 0.11 |
| T3 at 12-month follow-up (nmol/L) | 0.71 ± 0.22 | 0.77 ± 0.21 | 0.10 |
| fT4 at 12-month follow-up (nmol/L) | 1.23 ± 0.17 | 0.87 ± 0.14 | <0.001 |
| TSH at 12-month follow-up (mIU/L) | 3.10 ± 2.11 | 3.14 ± 2.24 | 0.90 |
| Baseline eGFR (mL/min/1.73m^2^) | 6.3 ± 5.3 | 5.0 ± 2.1 | 0.06 |
| Weekly KT/V | 2.3 ± 1.9 | 2.0 ± 0.7 | 0.09 |
| Peritoneal dialysis modality |  |  |  |
| CAPD | 90 (87.38) | 53 (84.13) | 0.56 |
| APD | 13 (12.62) | 10 (15.87) |  |
| Use of vitamin D | 16 (15.53) | 13 (20.63) | 0.40 |

Values are shown as mean ± standard deviation.

APD, automated peritoneal dialysis; CAPD, continuous ambulatory peritoneal dialysis; CRP, C-reactive protein; LDL, low-density lipoprotein; T3, triiodothyronine; TSH, thyroid-stimulating hormone
